# Incidence and Risk of Severe Ileus After Orthopedic Surgery: A Case-Control Study

**DOI:** 10.1007/s11420-019-09712-z

**Published:** 2019-08-16

**Authors:** Lisa A. Mandl, Mayu Sasaki, Jingyan Yang, Sara Choi, Kelianne Cummings, Susan M. Goodman

**Affiliations:** 1 Department of Rheumatology, Hospital for Special Surgery, 535 E. 70th Street, New York, NY 10021 USA; 2 Quality Research Center, Hospital for Special Surgery, 535 E. 70th Street, New York, NY 10021 USA; 3 Weill Cornell Medicine, 525 E. 68th Street, New York, NY 10021 USA; 4 Department of Biostatistics and Epidemiology, Hospital for Special Surgery, 535 E. 70th Street, New York, NY 10021 USA

**Keywords:** case–control studies, incidence, risk factors, orthopedics, ileus, diet, hospitalists

## Abstract

**Background:**

Post-operative ileus (POI) is common and can be associated with significant morbidity.

**Questions/Purposes:**

We aimed to identify the incidence of and risk factors associated with severe post-operative ileus (SPOI) after elective orthopedic surgery.

**Methods:**

We conducted a retrospective case–control study of patients undergoing elective orthopedic procedures at a single musculoskeletal specialty hospital. SPOI cases matched 1:2 to non-POI controls. International Classification of Diseases, Ninth Revision (ICD-9), codes were used to identify patients who were coded as having an episode of POI. After chart review, a subset was classified as clinical SPOI cases, based on set criteria. Regression models were constructed to identify variables associated with SPOI.

**Results:**

Of 273 POI cases, 77 (28.2%) were classified as SPOI. Overall rates of SPOI were 2.74/1000 orthopedic discharges, with SPOI most common in spine surgeries (9.07/1000 spine procedure discharges). Hypothesis-generating multivariable conditional logistic regression suggested that, for hip and knee cases, not being on a full diet by post-operative day (POD) 2 posed an increased risk of SPOI. For spine cases, not being on a full diet on POD 2 and longer surgery times were associated with risk of SPOI.

**Conclusions:**

In this retrospective case–control study, patients undergoing elective orthopedic procedures who had not progressed to full diet by POD 2 and spine patients with longer operative times were most at risk for SPOI. These data can be used clinically by peri-operative physicians to stratify patients according to risk.

**Electronic supplementary material:**

The online version of this article (10.1007/s11420-019-09712-z) contains supplementary material, which is available to authorized users.

## Introduction

Post-operative ileus (POI) has been reported in 0.7 to 4% of orthopedic surgery patients [11]. While transient ileus is a normal physiologic response to surgery, prolonged ileus is pathologic and can result in significant morbidity. Severe post-operative ileus (SPOI) is associated with an increase in venous thromboembolism, variability in dose response to warfarin, and even colonic perforation and death [2, 4, 9, 12]. In addition, patients may mobilize more slowly, leading to longer hospital stays and increased costs [10]. Although prior abdominal surgery, prolonged operative time, and prolonged opioid use have been reported as risk factors for POI in orthopedic surgery, these have been inconsistently described [10]. In addition, many studies do not differentiate between urgent and elective procedures; rates of SPOI may be lower when surgery is planned. Studies of ileus often rely exclusively on International Classification of Diseases (ICD) codes or chart review for the word “ileus” for case ascertainment; neither method differentiates clinically significant POI from normal post-operative bowel dysmotility.

The purpose of this study was to answer the following four questions: (1) What is the incidence of SPOI after elective orthopedic procedures? (2) What are the univariate associations of potential associated risk factors for SPOI after these procedures? (3) What are the multivariate associations of potential associated risk factors for SPOI after these procedures? (4) What are the adverse events associated with SPOI after these procedures?

## Patients and Methods

For this retrospective case–control study, all elective and nonemergent orthopedic procedures performed at a single musculoskeletal specialty hospital between March 2009 and April 2011 were eligible for inclusion. ICD-9 (Ninth Revision) codes (560.9, 997.4, 560.1, 560.89, 569.89) were used to identify the first post-operative episode of ileus during each admission, the index date being when the word “ileus” was first noted in the medical record. As there is no standard definition, three complementary methods were used to define SPOI: (1) a literature review, (2) an expert panel of three internists and a gastroenterologist, and (3) a review of all identified POI cases to understand the range of POI presentation in this patient population [1, 17]. SPOI was subsequently operationalized as a composite of the following: (1) patients with abdominal distention and no bowel movement by post-operative day 4, (2) administration of methylnaltrexone to prevent or mitigate severe ileus, (3) insertion of a nasogastric tube for the sole reason of preventing or mitigating severe ileus, (4) ileus diagnosed by a gastrointestinal (GI) consultant on or after post-operative day 4. After all cases of POI were identified, charts of all POI cases were reviewed by two investigators (K.C. and S.C.) to identify which met criteria for SPOI. Discrepancies were resolved by a third party (L.A.M. or S.M.G.).

Multiple potential risk factors for SPOI were considered based on literature review. Adverse events occurring prior to discharge were collected from the hospital chart.

SPOI cases were matched 1:2 to non-POI controls based on procedure type, procedure date, and procedure time of day (before or after noon). Procedure date was included to control for secular changes in hospital protocols. Patients progress to “diet as tolerated” as part of standard care for all surgical services, with no specific diet progression pathway. Time of the procedure was matched to ensure similar length of time that the patient status was NPO (nothing by mouth) prior to surgery. Because of inherent differences in approach and potential irritation of the peritoneum, cases and controls were grouped anatomically. Hip procedures included primary and revision total hip arthroplasty, hip arthrotomy, and open reduction of fracture with internal fixation. Knee procedures included primary and revision total knee arthroplasty, knee arthrodesis, arthroscopy, and open reduction of fracture with internal fixation. Spine procedures included primary and revision dorsal, dorsolumbar, lumbar, and lumbosacral decompression and fusion by anterior, posterior, and combined approaches. Upper extremity procedures were included as well.

Rates of SPOI were calculated per 1000 discharges. Overall summary statistics were reported in terms of means and standard deviations for continuous variables and frequencies and percentages for discrete variables. Descriptive statistics were performed using Fisher’s exact and *χ*^2^ tests for categorical variables, Student *t* tests for normally distributed continuous variables, and Mann–Whitney *U* test for non-normally distributed continuous variables. Univariate conditional logistic regression models were used to identify variables associated with SPOI. Two multivariable conditional logistic regression models were constructed to identify independent risk factors for SPOI: one for hip and knee cases (combined because of similar SPOI rates) and one for spine cases. Variables were selected for inclusion in the multivariable models if their univariate significance was < 0.05, with age and sex included a priori. A *p* value of < 0.05 in the multivariable models was considered statistically significant. Data were stored in REDCap (Research Electronic Data Capture, Vanderbilt University, Nashville, TN, USA) [8]. Clinically important pre-discharge adverse events (AEs) were identified via chart review. All analyses were carried out in Statistical Software (release 14; StataCorp, College Station, TX, USA). Institutional review board approval and a waiver of authorization were obtained prior to the start of study activities.

## Results

ICD-9 codes identified 273 POI cases, with “ileus” being first recorded in the chart by an orthopedic surgeon (12.1%), other physician (61.9%), nurse (7.3%), physician assistant (2.9%), nonphysician post-anesthesia care unit (PACU) staff (4.8%), or unidentified team member (6.2%). In the remaining 4.8% of cases, an ICD-9 code of ileus was attached to the admission due to ileus symptoms/treatments noted in the chart.

**Fig. 1:**
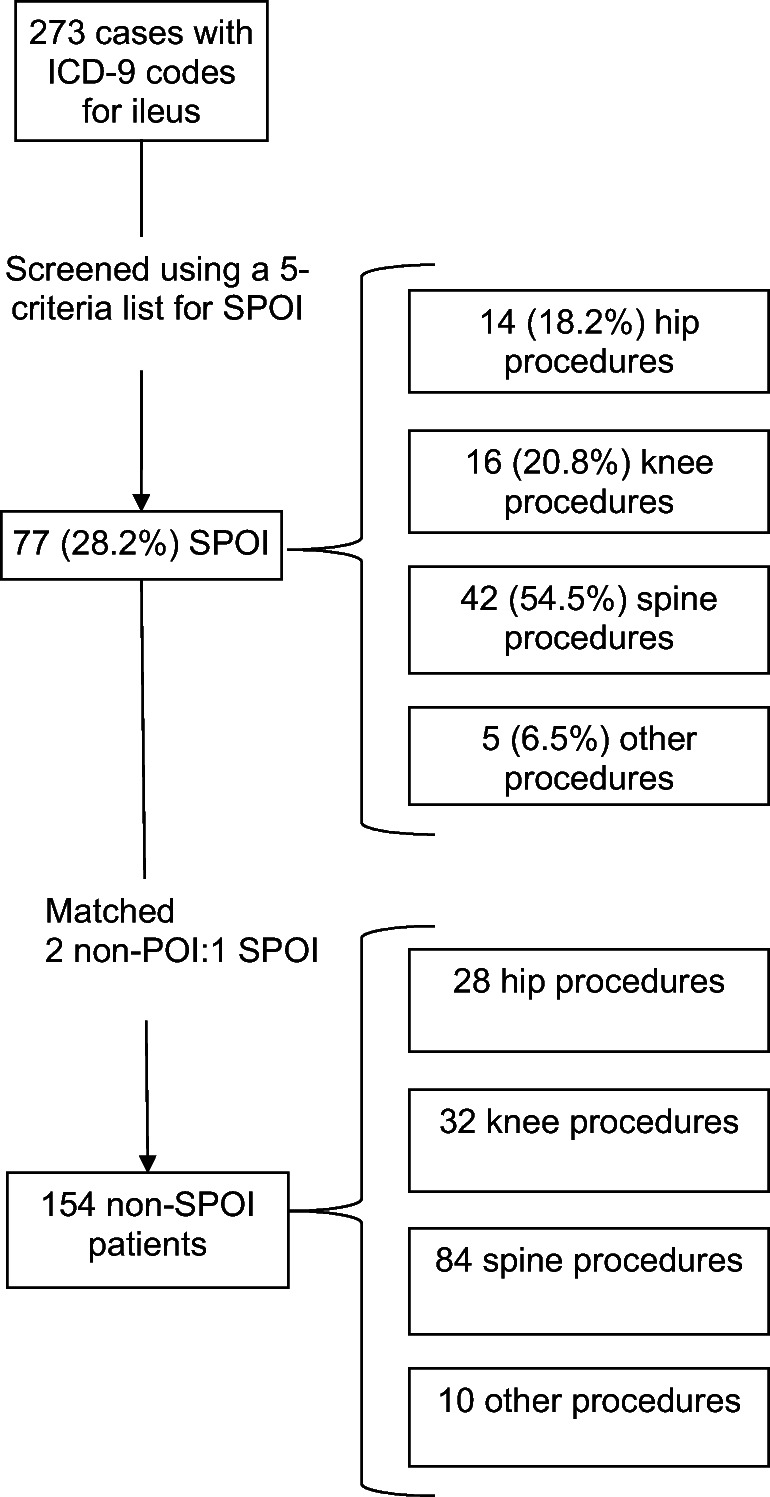
Study cohort. Number of severe post-operative ileus (SPOI) cases identified according to International Classification of Diseases, Ninth Revision (ICD-9) codes and controls matched within each procedure grouping.

**Fig. 2:**
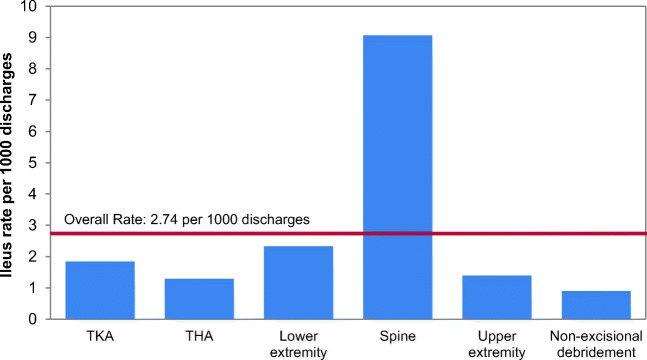
Ileus rates at the institution by procedure. TKA (total knee arthroplasty) includes both primary and revision procedures, and THA (total hip arthroplasty) includes both primary and revision procedures. Arthroplasty cases are reported separately to facilitate cross-institutional comparisons.

Of all POI cases, 28.2% (77/273) met the definition of SPOI: 18.2% (14) were hip procedures, 20.8% (16) were knee procedures, 54.5% (42) were spine procedures, and 6.5% (5) were other orthopedic procedures (Fig. 1). The overall rate of SPOI was 2.74/1000 discharges. Rates were highest for spine (9.07/1000 discharges) and lowest for upper extremity procedures (1.40/1000 discharges) (Fig. 2). Patients with SPOI after hip procedures were older than controls, more likely to be male, and have higher body mass index (BMI). Knee SPOI patients were also more likely to be male. Values for age, sex, and BMI were similar between patients with SPOI after spine procedures and controls. Patients with SPOI after hip and knee procedures were also more likely to be on anti-cholinergic medications pre-operatively, compared with patients who did not develop SPOI (Tables 1, 2, 3).

**Table 1:** Hip patient demographics and study population

	Case (mean ± SD or *n*, %)	*n*	Control (mean ± SD or *n*, %)	*n*	*p* value
Age (years)	75.4 ± 7.5	14	64.4 ± 17.7	28	0.03
≥ 60 years	14 (100.0)	14	17 (60.7)	28	0.007
Female	2 (24.3)	14	13 (46.4)	28	0.04
Body mass index (BMI)	33.4 ± 7.7	14	28.3 ± 7.0	28	0.04
Overweight/obese (BMI ≥ 25)	14 (100.0)	14	15 (53.6)	28	0.002
Length of stay (days)	13.4 ± 8.3	14	6.8 ± 9.8	28	0.04
Length of surgery (h)	2.1 ± 1.1	13	1.8 ± 0.8	27	0.44
Total PCA (Dilaudid) delivered via infusion 24 h prior to first mention of ileus (μg)	1820.0 ± 0.0	1	136.0 ± 240.5	10	< 0.001
Total PCA attempts 24 h prior to first mention of ileus	6.0 ± 0.0	1	0.6 ± 0.8	10	< 0.001
Received Dilaudid via PCA 24 h prior to first mention of ileus	1 (7.1)	14	10 (35.7)	28	0.07
Pre-admission medications					
Iron supplement	0 (0.0)	14	5 (17.9)	28	0.09
Proton pump inhibitor	3 (21.4)	14	6 (21.4)	2828	1.00
Calcium channel blockers	5 (35.7)	14	4 (14.3)	28	0.11
Calcium supplements	0 (0.0)	14	6 (22.2)	27	0.06
Opioids	0 (0.0)	14	9 (32.1)	28	0.02
Anti-cholinergics	2 (14.3)	14	0 (0.0)	28	0.04
Constipation	4 (28.6)	14	0 (0.0)	28	0.003
Diarrhea	2 (14.3)	14	0 (0.0)	28	0.04
Diverticulitis	2 (14.3)	14	0 (0.0)	28	0.04
Previous abdominal surgery	8 (57.1)	14	12 (42.9)	28	0.38
Transfusion prior to date ileus first mentioned	1 (14.3)	7	0 (0.0)	0	NA
Diet on post-operative day 2					
NPO	3 (21.4)	14	0 (0.0)	28	0.03
Clear fluids	1 (7.1)	14	2 (7.1)	28	
Soft foods	3 (21.4)	14	3 (10.7)	28	
Full diet	6 (42.9)	14	23 (82.1)	28	
Multiple surgeries in the same admission	3 (21.4)	14	1 (3.6)	28	0.10
Highest pain score 2 days before ileus first mentioned (10-point visual analogue scale)	4.9 ± 3.6	14	6.1 ± 3.3	28	0.32

*PCA* patient-controlled analgesia, *NPO* nothing by mouth

No cases or controls had a history of radiation to the thorax

**Table 2:** Knee patient demographics and study population

	Case (mean ± SD or *n*, %)	*n*	Control (mean ± SD or *n*, %)	*n*	*p* value
Age (years)	63.1 ± 13.3	16	68.7 ± 10.3	32	0.14
≥ 60 years	14 (87.5)	16	20 (62.5)	32	0.10
Female	4 (25.0)	16	17 (53.1)	32	0.08
Body mass index (BMI)	31.8 ± 9.1	16	30.4 ± 4.9	32	0.59
Overweight/obese (BMI ≥ 25)	14 (87.5)	16	26 (81.3)	32	0.70
Length of stay (days)	20.6 ± 47.2	16	3.9 ± 1.4	32	0.05
Length of surgery (h)	1.7 ± 0.7	15	1.7 ± 0.8	32	0.97
Total PCA (Dilaudid) delivered via infusions 24 h prior to the first mention of ileus (μg)	269.8 ± 243.9	6	1610.8 ± 3103.7	11	0.31
Total PCA attempts 24 h prior to the first mention of ileus	3.7 ± 6.2	6	11.3 ± 13.9	12	0.23
Received any PCA Dilaudid 24 h prior to the first mention of ileus	6 (37.5)	16	11 (34.4)	32	0.83
Pre-admission medications					
Iron supplement	5 (15.6)	16	2 (12.5)	32	1.00
Proton pump inhibitor	7 (43.8)	16	8 (25.5)	32	0.19
Calcium channel blockers	3 (18.8)	16	7 (21.9)	32	1.00
Calcium supplements	2 (12.5)	16	11 (34.4)	32	0.17
Opioids	4 (25.0)	16	3 (9.4)	32	0.20
Anti-cholinergics	4 (25.05)	16	0 (0.0)	32	0.009
Constipation	2 (12.5)	16	1 (3.1)	32	0.25
Diverticulitis	1 (6.3)	16	2 (6.3)	32	1.00
Previous abdominal surgery	10 (62.5)	16	19 (59.4)	32	1.00
Transfusion prior to date ileus first mentioned	1 (75.0)	4	0 (0.0)	0	NA
Any other adverse events	0 (0.0)	16	0 (0.0)	32	NA
Diet on post-operative day 2					
NPO	2 (12.5)	16	0 (0.0)	31	0.05
Clear liquids	1 (6.3)	16	0 (0.0)	31	
Soft foods	2 (12.5)	16	4 (12.9)	31	
Full diet	8 (50.0)	16	26 (83.9)	31	
Multiple surgeries in the same admission	1.0 ± 6.3	16	0.0 ± 0.0	32	0.33
Highest pain score 2 days before ileus first mentioned (10-point visual analogue scale)	6.0 ± 3.6	16	5.9 ± 3.4	32	0.91

*PCA* patient-controlled analgesia, *NPO* nothing by mouth

No cases or controls had a history of radiation to the thorax

**Table 3:** Spine patient demographics and study population

	Case (mean ± SD or *n*, %)	*n*	Control (mean ± SD or *n*, %)	*n*	*p* value
Age (years)	54.0 ± 16.7	42	56.2 ± 16.0	84	0.47
≥ 60	16 (38.1)	42	34 (40.5)	84	0.85
Female	21 (50.0)	42	45 (53.6)	84	0.71
Body mass index (BMI)	25.9 ± 6.2	42	26.4 ± 4.7	84	0.65
Overweight/obese (BMI ≥ 25)	20 (47.6)	42	45 (53.6)	84	0.57
Length of stay (days)	12.4 ± 8.4	42	5.5 ± 3.0	84	< 0.001
Length of surgery (h)	6.4 ± 2.7	41	4.8 ± 2.1	84	0.002
Total PCA opioids (Dilaudid and morphine) delivered via infusion 24 h prior to the first mention of ileus (μg)	9176.7 ± 8159.2	21	7175.9 ± 13614.8	27	0.56
Total PCA attempts 24 h prior to the first mention of ileus	27.6 ± 16.6	21	16.0 ± 13.6	27	0.01
Received opioids (Dilaudid and morphine) 24 h prior to the first mention of ileus	20 (47.6)	42	27 (32.1)	84	0.17
Pre-admission medications					
Iron supplements	8 (19.0)	42	13 (15.5)	84	0.61
Proton pump inhibitor	13 (31.0)	42	18 (21.4)	84	0.24
Calcium channel blocker	8.0 (19.0)	42	21 (25.0)	84	0.45
Calcium supplements	11 (26.2)	42	19 (22.6)	84	0.07
Opioids	19 (45.2)	42	39 (46.4)	84	1.00
Anti-cholinergic use	9 (21.4)	42	8 (9.5)	84	0.07
Constipation	9 (21.4)	42	13 (15.5)	84	0.41
Diverticulitis	3 (3.6)	42	2 (4.8)	84	0.41
Previous abdominal surgery	16 (38.1)	42	33 (39.3)	84	0.9
Transfusion prior to date ileus first mentioned	8 (53.3)	15	0 (0.0)	0	NA
Any other adverse events	6 (14.3)	42	0 (0.0)	84	0.04
Diet on post-operative day 2					
NPO	8 (20)	40	2 (2.5)	79	< 0.001
Clear liquids	14 (35.0)	40	9 (11.4)	79	
Soft foods	2 (5.0)	40	7 (8.9)	79	
Full diet	10 (25.0)	40	52 (65.8)	79	
Multiple surgeries in the same admission	3.0 ± 7.1	42	1.0 ± 1.3	84	0.11
Highest pain score 2 days before ileus first mentioned (10-point visual analogue scale)	7.7 ± 2.9	42	6.6 ± 2.6	84	0.02

*PCA* patient-controlled analgesia, *NPO* nothing by mouth

No cases or controls had a history of radiation to the thorax

Univariate conditional logistic regression models for hip and knee cases found associations between SPOI and an age of 60 years or older (odds ratio [OR], 12.9; 95% confidence interval [CI], 1.68–99.12; *p* = 0.014), a BMI of 25 or higher (OR, 6.72; 95% CI, 1.45–31.02; *p* = 0.015), constipation (OR, 12.0; 95% CI, 1.44–99.67; *p* = 0.021), and not being on a full diet by post-operative day 2 (OR, 3.84; 95% CI, 1.33–11.14; *p* = 0.013). Pre-admission calcium supplements were protective (OR, 0.20; 95% CI, 0.04–0.89; *p* value = 0.035), as was being female (OR, 0.19; 95% CI, 0.05–0.66; *p* value 0.009). Similar analyses for spine cases showed longer surgery (OR, 1.77; 95% CI, 1.32–2.38; *p* value < 0.001), higher pain scores 2 days prior to ileus (OR, 1.18; 95% CI, 1.02–1.37; *p* value = 0.028), and not being on a full diet by post-operative day 2 (OR, 9.83; 95% CI, 2.88–33.72; *p* value < 0.001) increased the risk of SPOI.

Two multivariable conditional logistic regression models were constructed, one for hip and knee combined, and one for spine. Due to the small number of SPOI cases when stratified by procedure, these should be viewed as hypothesis generating. Age and sex were included a priori. In addition, based on the univariate analysis, pre-operative anti-cholinergic use was included in the multivariable analysis for the spine model. Controlling for age, sex, BMI, calcium supplements prior to admission, constipation, the hip and knee model showed that not being on a full diet by post-operative day 2 increased the risk of SPOI (OR, 7.96; 95% CI, 1.17–53.94; *p* value = 0.034). For spine cases, after controlling for age, sex, BMI, and taking anti-cholinergic medication prior to admission, not being on a full diet by post-operative day 2 was also associated with a higher risk of SPOI (OR, 11.70; 95% CI, 1.81–75.49; *p* value = 0.010), as were longer surgery times (OR, 2.14; 95% CI, 1.16–3.97; *p* value = 0.015). Taking anti-cholinergic medications prior to admission was non significantly associated with SPOI (OR, 6.62; 95% CI, 0.75–58.20; *p* value = 0.09).

Four AEs (acute pancreatitis, atrial fibrillation, peripheral nerve injury, and a urinary tract infection; 9.5%) occurred in patients with SPOI after spine procedures; none occurred in spine controls (*p* < 0.001). Four AEs occurred in two patients with SPOI after hip procedures (atrial fibrillation, ventricular tachycardia, *Clostridium difficile* infection, and death; 14.2%) and none in hip controls (*p* = 0.040). There were no AEs in any patients after knee procedures (either SPOI cases or controls).

## Discussion

The aim of this study was to identify risk factors for severe ileus after a range of nonemergent orthopedic procedures. These patients are commonly managed post-operatively by a hospitalist rather than their operating surgeon, and thus it is important that both hospitalists and orthopedic surgeons have reliable information on which patients may be at highest risk for SPOI. An important observation is that 71.8% of cases with ICD-9 codes for ileus did not meet our rigorous definition of physiologically significant ileus. This has important implications, as ileus rates are used as actionable hospital quality measures and are likely to be incorrectly inflated if only ICD codes are used for case identification [17]. We found that documentation of ileus, thereby triggering the attachment of an ICD-9 ileus code, was done by a variety of healthcare professionals. Therefore, it is important that efforts to improve charting accuracy not be directed only at physicians.

There are several limitations to this study. Our cases were from 2009 to 2011, and since that time, there may have been secular changes in operative and peri-operative care of orthopedic patients, which may affect rates of SPOI. In particular, responses to the opioid epidemic have led to significant decreases in the use of post-operative opioids for pain management. Although we did not find a relationship between opioids and severe ileus in our study, this relationship should be explored in a contemporary cohort. As in all retrospective studies, there is the possibility of unmeasured confounding, but our use of matched controls from the same surgical population was a means of mitigating this potential source of bias. Over-the-counter (OTC) drug use was not systematically examined, and therefore, anti-cholinergic OTC drugs may have been missed. Thus, the effect size of anti-cholinergic use may be larger than we observed in this study. Also, since there were only relatively small numbers of SPOI cases when stratified by procedure, our confidence intervals are wide, and our multivariable regressions should be considered exploratory.

There are also several strengths to this study. We defined SPOI based on an expert’s input, a literature review, and clinical presentation in the specific study population, not just ICD-9 codes [17]. Risk factors were validated with double chart review. We excluded emergent procedures to ensure generalizability, as trauma and other emergent cases may have different risk associations.

In contrast to previous studies, we found older age to increase the risk of SPOI in patients undergoing hip or knee procedures [1]. This may reflect our more stringent definition of severe ileus, our inclusion of all hip and knee procedures and not just joint arthroplasty, and our use of a control group consisting of patients without ileus rather than mild ileus. Being overweight or obese also increased the risk of SPOI in hip and knee patients. This may relate to the need for more strenuous manipulations to gain clear visualization of the surgical field or perhaps to a larger volume of distribution of anti-cholinergic anesthetic medications in the adipose tissue, with subsequent prolonged bowel dysmotility. Similar to previous studies, we found that being female decreased the risk of SPOI [1, 7, 14, 15]. The reasons for this are not known, though in general, women have a lower BMI than men. Use of pre-operative calcium supplements decreased risk of SPOI, whereas a history of constipation increased the risk. This counterintuitive finding likely reflects confounding by indication, with patients declining to take calcium if they are already prone to constipation. Systematically inquiring about constipation in patients pre-operatively may help identify those at increased risk for SPOI.

For spine cases, each increased hour in surgery increased the odds of SPOI by 77%. This suggests that patients with lengthy procedures should be monitored carefully post-operatively. In addition, higher pain scores prior to ileus were associated with an increased risk of SPOI. While there were no differences detected in the amount of opioids administered by patient-controlled analgesia, inadequately treated pain could contribute to the release of vasoactive hormones, increasing the risk of ileus [5].

The trend toward higher pre-operative anti-cholinergic use among patients who develop SPOI is intriguing. The use of medications with anti-cholinergic effects is highly prevalent [13]. Anti-cholinergic medications block the parasympathetic nervous system, responsible for the involuntary muscle activity of the gastrointestinal tract. They are therefore a potentially modifiable risk factor for ileus, and thoughtful discontinuation pre-operatively may decrease the risk of ileus. This would include commonly used OTC therapies such as Advil® PM, Alev® PM, and Tylenol® PM that contain diphenhydramine and are not usually asked about pre-operatively.

We have additionally identified slow advancement of post-operative feeding as a factor strongly associated with SPOI, with patients still NPO on post-operative day 2 at significantly increased risk of SPOI. This study was retrospective, and delayed feeding was one of our criteria for SPOI, and so whether there is a causal link cannot be established. However, early post-operative feeding has been found to decrease the rates of ileus, and studies evaluating more aggressive refeeding in elective orthopedic patients should be undertaken [3, 6, 16]. Regardless, slow refeeding is an easy-to-identify red flag for SPOI.

We found very few serious adverse events associated with SPOI. This is encouraging and suggests decreased morbidity due to SPOI, compared with older reports [2, 9, 14] and may reflect general improvements in peri-operative care.

We have identified risk factors for SPOI in patients undergoing common elective orthopedic procedures, for whom higher vigilance is warranted. For hip and knee patients, being older, being male, and having a BMI of 25 or higher increase the risk. In spine patients, longer procedure times and higher pain levels increase the risk.

Importantly, we have identified the pre-operative use of anti-cholinergic medications as a possible novel modifiable risk factor for SPOI. In addition, a history of constipation may also increase risk. Finally, not being on a full diet by post-operative day 2 using standard diet advancement protocols is associated with SPOI in all cases. These observations can be used to inform policies for monitoring patients after elective orthopedic surgeries. With the aging population, the number of elective orthopedic procedures is anticipated to rise dramatically. Patients undergoing elective orthopedic procedures are often not as ill as hospitalized medical patients, and busy hospitalists caring for a broad orthopedic case mix may not be attuned to the risks of potential SPOI in these less acutely ill patients. Therefore, having easily identifiable red flags for SPOI should be clinically useful and help to optimize outcomes for this growing group of patients.

## Electronic supplementary material

ESM 1 (PDF 1224 kb)
